# The Amendment Bill

**Published:** 1913-08-16

**Authors:** 


### OUR NATIONAL INSURANCE ACT DEPARTMENT.

### LXIV.—The Amendment Bill.

The Amendment Bill to the National Insurance Act of 1911 has now passed into its third edition, and is ready for the consideration of the House of Lords. In its present shape it does not differ very materially from that in which it emerged after consideration by Standing Committee C. There are, however, a few alterations of importance to be noted. Two new clauses have been added and several new sub-sections have also found places in the Bill for the first time.

### Possible Alternative to the Panel System.

Of the two new clauses the one which is of the greatest importance is that by which alternative arrangements for the panel system are to be allowed in certain circumstances. The first of the points to be noted in this new clause is that the Insurance Commissioners must be satisfied that the insured persons are not receiving satisfactory medical treatment under the panel system.

This probably means that when complaints arise an inquiry will be instituted to determine the facts, and also possibly to suggest the means of rectification if the panel system is shown to be defective. Secondly, the new powers given to the Insurance Commissioners by the clause are not intended for the exceptional case of individual persons desirous of making their own arrangements when there is a satisfactory panel, but are designed to meet the case of general discontent with the panel. For this reason the clause is drafted to provide that when a *considerable proportion* of the insured persons in a Committee area, or a part of a Committee area, are not receiving satisfactory treatment under the existing panel system the alternative arrangements shall be brought into operation. It will thus be seen that local circumstances are to be taken into account. This is, of course, as it should be when such an important matter in regard to the public health of the locality as the medical treatment of insured persons is in question.

On the other hand, it may be admitted that the way is opened up for a piecemeal destruction of the panel system, and it is very possible that, if the clause is finally passed in its present shape, attempts will be made at an early date either by the doctors or by the insured persons, or perhaps by both, in a large number of districts to obtain a substitute for the present panel system.

Another point to be noted is that, whereas under the existing Act the Insurance Committees have practically a free hand in making the arrangements for the administration of medical benefit provided such arrangements comply with the regulations of the Insurance Commissioners, under the new clause the Commissioners, if satisfied as to the necessity for such action, may authorise the Committee to make other arrangements, or the Commissioners may take the matter out of the hands of the Committee and make the arrangements themselves. In either case the substituted arrangements for the better medical service will be strictly limited by the amount of the funds available for the purpose, and it must not be supposed that the alteration in the machinery for the administration of medical benefit will result, in the circumstances, in any substantial enlargement of the facilities for obtaining medical treatment and attendance.

If it be not feasible for either the Insurance Committee or the Insurance Commissioners to make other arrangements, as, for example, by the appointment of whole-time medical officers for attendance on insured persons, the insured persons may be called upon to make their own arrangements. In this event the Insurance Committee or the Commissioners will have to undertake to pay the cost of treatment and attendance, including medicines, according to a prescribed scale. The calculation of this scale is to allow for attendance and treatment not inferior to that provided under the panel system. Presumably, the additional treatment desired over and above that obtainable under the panel system, which will be the reason for dissatisfaction with that system, will have to be paid for by the insured persons out of their own resources.

### "Inmates" of Charitable Institutions.

The other new clause is of considerable importance to those who are interested in institutions carried on for charitable or reformatory purposes. Under the principal Act it is possible for the managers of such institutions to take advantage of a section which exempts them from the necessity of stamping Insurance cards for their *inmates* in certain circumstances. The word *inmate* has apparently been a matter of contention, for the provisions of the section alluded to are now to apply only to such persons who are inmates of a charitable or reformatory institution for the purposes for which the institution exists.

Thus, nurses, staff, and attendants are excluded from the operation of the special provisions relating to these institutions in the principal Act.

The word "inmate" is used in a similar way in Section 12 of the National Insurance Act, which precludes the payment of sickness and disablement benefits to insured persons who are inmates of hospitals. In this case also it will be remembered that the use of the word gave rise to difficulty, and it was only when the Insurance Commissioners formally decided that the general staff of a hospital were not for this purpose to be considered inmates of the hospital that the way was made clear for them to receive the benefits for which they had paid. It seems to us, therefore, that the new clause is either not wanted or else that it does not go far enough.

If the Commissioners were justified in deciding as to the interpretation to be placed on the word "inmate" as used in Section 12, by virtue of their power to remove difficulties, they surely have the same power in regard to charitable and reformatory institutions. If, on the other hand, they were acting *ultra vires* in giving that decision, the interpretation of the word, in the section where it is of much greater significance than in relation to charitable and reformatory institutions, should not be left to chance, and the new clause should be extended accordingly.

### Maternity Benefit to be the Mother's.

The principal discussion in Committee on new clauses to the Amendment Bill as originally introduced centred round the provision of maternity benefit. We dealt with this subject at some length in our last week's issue. The matter is one of great importance, as the proper administration of this benefit is calculated to have an immense influence for good not only on the mothers of the present generation, but also on the health of the next and future generations.

The main contention of the new clause, added by the Committee to the Bill—namely, that the maternity benefit is in all cases to be the mother's benefit—was confirmed on the Report stage, and forms part of the Bill as it now proceeds for the consideration of the House of Lords.

Very substantial alterations were, however, made in the drafting of the clause. The Government left the members free to vote irrespective of the Party Whips, and it may therefore be said that the clause as it at present stands expresses the unbiassed opinion of the majority of the House. At the same time it might perhaps have been well if Mr. Masterman had given a clearer lead, for it is supposed that the last amendment, proposed by Lord Robert Cecil and carried, may have the effect of increasing the difficulties of the approved societies in administering the benefit and may in a large number of cases cause delay in payment, which, however, the societies must learn to avoid.

Under the principal Act maternity benefit is paid from the husband's insurance, when he is insured, and his receipt, except in special circumstances, is required to discharge the obligation of his society. Now it is proposed that if the maternity benefit is paid from the husband's insurance "the wife's receipt or his receipt, *if authorised by her*, on her behalf shall be a sufficient discharge." Here, then, is the position. In the vast majority of cases the maternity benefit comes from the husband's insurance. The husband is precluded from giving a valid discharge to his society unless he holds an authority from his wife enabling him to do so. For some time after the birth of the child the wife may no more be able to sign an authority to pay her husband than to sign a direct receipt for payment to herself. This may at first mean delay in obtaining payment, for the approved societies have to be able to produce to the Government auditors receipts and vouchers for all payments, and the risk of a payment being disallowed is not one they care to take.

Furthermore, the approved societies—particularly those of the centralised type—will experience a difficulty because they will be called upon to make a payment on a signature which they have no means of testing till a way be found.

In view of the wording towards the end of the same sub-section that the benefit is to be "administered in the interests of the mother and child," coupled with the penal provisions of the Act that can be used in extreme cases of misappropriation of the benefit, it appears to us that it would have been better to have left the clause as it stood on passing the Standing Committee—namely, that the receipt of the wife alone would be sufficient discharge.

### Payment of Benefit on Leaving Hospital.

The amendment of Section 12 of the National Insurance Act, whereby payments of benefit are regulated when the insured person is in hospital, has already been alluded to in our columns. The clause of the Bill which was inserted in Committee remains substantially as when it was introduced. An effort was made, however, to require the payment to the hospital of a substantial proportion of the benefit, when paid in cash upon leaving, towards the cost of maintenance and treatment of the insured person while he was an inmate. The question only arises when the insured person has no dependants, for when there are dependants the benefit is paid to them. In the circumstances, Parliament decided that it was inadvisable to compel the insured person to contribute to the hospital, and the matter has been left to the voluntary action of each individual patient who may be so treated.

Thus, while the voluntary principle is preserved, the hospital managers will still be faced with the problem of how best to find the money for treating insured persons, many of whom are now to be allowed to go away with a substantial cash sum derived from their approved societies. While the new clause is undoubtedly an improvement upon the section of the principal Act, it cannot be accepted as a final settlement of the difficulty, and some other means must be sought before the next Amendment Bill is drafted.

### A Minor Defect Corrected.

The opportunity afforded by the Amendment Bill was taken to amplify the powers of the Scottish Commissioners, which were shown to be defective after the Bill had been introduced. It was decided in the Courts that they had no power to recover unpaid contributions summarily as a civil debt. This power has now been given them, and they are now on the same footing as the English Commissioners in this respect.

